# Right ventricular strain predicts outcome in patients receiving sacubitril/valsartan: A sub‐analysis of DISCOVER‐ARNI

**DOI:** 10.1002/ehf2.15297

**Published:** 2025-04-16

**Authors:** Maria Concetta Pastore, Giulia Elena Mandoli, Alberto Giannoni, Francesca Rubina Ginetti, Michele Correale, Natale Daniele Brunetti, Andrea Igoren Guaricci, Laura Piscitelli, Anna Degiovanni, Giuseppe Patti, Alessandro Malagoli, Luca Moderato, Erberto Carluccio, Paolo N. Marino, Michele Emdin, Matteo Cameli

**Affiliations:** ^1^ Department of Medical Biotechnologies, Division of Cardiology University of Siena Siena Italy; ^2^ Cardiology and Cardiovascular Medicine Department Fondazione Toscana G. Monasterio Pisa Italy; ^3^ Institute of Life Sciences Scuola Superiore Sant'Anna Pisa Pisa Italy; ^4^ Cardiology Department Policlinico Riuniti University Hospital Foggia Italy; ^5^ Department of Medical and Surgical Sciences University of Foggia Foggia Italy; ^6^ Interdisciplinary Department of Medicine, University Cardiology Unit A.O.U.C. Polyclinic of Bari Bari Italy; ^7^ Department of Thoracic, Heart and Vascular Diseases Maggiore della Carità Hospital Novara Italy; ^8^ Department of Translational Medicine University of Piemonte Orientale Novara Italy; ^9^ Division of Cardiology, Nephro‐Cardiovascular Department Baggiovara Hospital University of Modena and Reggio‐Emilia Modena Italy; ^10^ Cardiology Department Ospedale Guglielmo da Saliceto – Piacenza Piacenza Italy; ^11^ Cardiology and Cardiovascular Pathophysiology ‐ Heart Failure Unit “Santa Maria della Misericordia” Hospital University of Perugia Perugia Italy; ^12^ Istituto Iperbarico Villafranca di Verona Verona Italy

**Keywords:** Sacubitril/valsartan, Right ventricular, Strain, Speckle tracking, Heart failure, Prognosis

## Abstract

**Aims:**

Angiotensin receptor/neprilysin inhibitors (ARNI) have emerged as a pivotal medical treatment considerably improving the clinical outcome of patients with heart failure and reduced ejection fraction (HFrEF). Identifying individuals who stand to benefit the most from ARNI could markedly enhance patient management strategies. The aim of this sub‐analysis of DISCOVER‐ARNI register was to evaluate the prospective prognostic significance of speckle tracking echocardiography (STE) parameters in patients undergoing ARNI therapy.

**Methods and results:**

DISCOVER‐ARNI multicentre Italian register retrospectively enrolled 341 patients with HFrEF referred for treatment with ARNI. These patients underwent clinical, biohumuoral, and echocardiographic assessment at baseline. Subsequently, among those with available right ventricular STE data, a prospective long‐term follow‐up was conducted by telephone interview or on‐site visits. The primary endpoint encompassed a composite of outcomes, including all‐cause or cardiovascular mortality, heart failure hospitalization, heart transplantation, and left ventricular assist device (LVAD) implantation. Overall, 136 HFrEF patients were included in this sub‐analysis (mean age 65 ± 10 years, 82% male). The mean follow‐up was 40 ± 18 months, during which 32 patients reached the primary endpoint (14 deaths of which 10 due to cardiovascular reasons, 22 hospitalization, 3 heart transplantation, 1 LVAD implantation). Baseline assessment revealed that patients with events had higher LV volumes and EF (LV end‐diastolic volume 212 ± 65 vs.174 ± 57 mL, *P = 0.002*; LV end‐systolic volume 156 ± 52 vs. 122 ± 49 mL, *P = 0.001*; LV EF = 26 ± 5 vs. 29 ± 5 mL, *P = 0.006*, respectively), lower but preserved tricuspid annular plane systolic excursion (TAPSE, 17 ± 3 vs.19 ± 3, *P = 0.008*), and higher systolic pulmonary artery pressures (38 ± 11 vs. 31 ± 8 mmHg, *P = 0.001*) compared to those who did not experience events. LV, left atrial (LA), and free wall right ventricular longitudinal strain (fwRVLS) were reduced in patients with events (−7 ± 2 vs. −8 ± 2%, *P = 0.002*; 11 ± 3 vs. 15 ± 7%, *P = 0.001* and −15 ± 5 vs. −22 ± 5%, *P = 0.007*, respectively). Employing Cox proportional hazard model including LVEF, TAPSE, RVFAC, LV strain, LA strain, and fwRVLS, the latest emerged as the sole independent predictor of the combined endpoint (hazard ratio = 1.15 [1.05;1.26], *P* = 0.002). Receiver operating characteristic (ROC) curves determined that fwRVLS = −20% was the optimal cut‐off for predicting the combined endpoint (area under curve [AUC] = 0.70). This threshold was used for constructing Kaplan–Meier survival curves, demonstrating effective risk stratification of fwRVLS over long‐term follow‐up for the primary endpoint.

**Conclusions:**

fwRVLS by STE holds promise as a valuable parameter to assess response to ARNI therapy in terms of overall survival, heart failure hospitalizations, heart transplantation, or LVAD implantation.

## Graphical abstract

Angiotensin receptor/Neprilysin inhibitors (ARNI) have shown to considerably improve the clinical outcome of patients with heart failure and reduced ejection fraction (HFrEF). Identifying individuals who may benefit from ARNI the most could markedly enhance patient management. In this sub‐analysis of DISCOVER ARNI study conducted over 136 HFrEF patients, free wall right ventricular longitudinal strain (fwRVLS)>‐20% emerged as the sole independent predictor of a combined endpoint including all‐cause or cardiovascular mortality, HF hospitalization, heart transplantation, left ventricular assist device (LVAD) implantation.

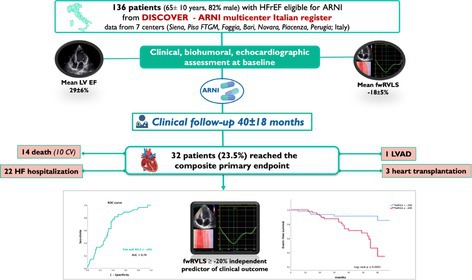

## Introduction

Sacubitril/valsartan represents the first‐in‐class angiotensin receptor/neprilysin inhibitor (ARNI). ARNIs have revolutioned the treatment paradigm for heart failure with reduced ejection fraction (HFrEF), owing to its favourable impact on morbidity and mortality, partly attributed to left ventricular (LV) reverse remodelling.^1^, ^2^, ^3^, ^4^ However, despite being recommended as an alternative to ACE inhibitors by European guidelines (class IB),^5^ many clinicians remain hesitant to prescribe ARNI as first‐line therapy.^6^, ^7^ Identifying individuals who would derive the greatest benefit from ARNI therapy could enhance patient selection for these medications.

Echocardiography serves as a first‐line imaging modality for evaluating patients with heart failure (HF). Two‐dimensional (2D) strain imaging techniques, such as speckle tracking echocardiography (STE), offer novel structural and functional indices for assessing cardiac chambers, comparable to invasive haemodynamic parameters.^8^ The significance of STE parameters is emerging in the clinical decision‐making, particularly for patient selection for initiating therapy and prognostication of patients with HF. Regarding LV analysis, LV global longitudinal strain (GLS) has emerged as an indicator of myocardial fibrosis and, consequently, an independent predictor of all‐cause mortality, surpassing other echocardiographic parameters in HFrEF patients.^8^, ^9^, ^10^ Peak atrial longitudinal strain (PALS) has proven sensitive in estimating LV filling pressures, therefore serving as an indicator of left atrial (LA) diastolic function,^8^ comparable to the degree of LA ultrastructural modifications. Indeed, both LV GLS and PALS have emerged as sensitive and reliable markers of cardiac remodelling and function,^11^ providing incremental prognostic information beyond standard echocardiographic parameters, influencing clinical and therapeutic management.

Over the past decade, the study of the right ventricle (RV) has gained prominence in the prognostic assessment of HFrEF patients, mainly with advances in new treatments, such as ventricular assist devices.^12^, ^13^ RV dysfunction signals progression to advanced biventricular HF and portends a poor prognosis.^13^, ^14^, ^15^, ^16^ Therefore, meticulous echocardiographic analysis of this cardiac chamber^9^ structure and function is crucial, as adverse events and mortality escalate with declining RV function.^13^ Indeed, RV function has emerged as a robust predictor of mortality in several cardiovascular disease, notably HF.^12^ STE offers greater sensitivity in assessing RV function in HFrEF patients, providing additional prognostic insights.^14^, ^16^, ^17^ Therefore, our aim was to analyse the potential prognostic value of STE parameters in a cohort of HFrEF patients undergoing ARNI therapy, utilizing data from a multicentre Italian registry named Deformation Imaging by Strain in Chronic Heart Failure Over Sacubitril‐Valsartan: A Multicenter Echocardiographic Registry (DISCOVER)‐ARNI.^1^

## Methods

The DISCOVER‐ARNI multicenter Italian registry retrospectively enrolled 341 patients with HFrEF eligible for ARNI therapy from 13 Italian centres. Clinical, biohumoural, and echocardiographic parameters were assessed at baseline and after 6 months of therapy. The DISCOVER‐ARNI investigated predictors of LV remodelling with specific emphasis on STE parameters. Furthermore, a sub‐analysis^18^ was published demonstrating the persistence of indication for implantable cardioverter‐defibrillator (ICD) implantation after 6 months of ARNI therapy.

In this observational study, we performed a sub‐analysis by prospectively collecting long‐term follow‐up data, either through telephone interview or on‐site visits, regarding the clinical outcomes of patients enrolled in DISCOVER‐ARNI and with available STE data for LV, LA, and RV. These data were only available from specific centres: ‘Le Scotte’ University Hospital in Siena, ‘G. Monasterio’ Tuscany Foundation in Pisa, Polyclinic ‘Riuniti’ University Hospital in Foggia, Polyclinic University Hospital in Bari, ‘Maggiore della Carità’ Hospital in Novara, ‘Guglielmo da Saliceto’ Hospital in Piacenza, and ‘Santa Maria della Misericordia’ Hospital in Perugia.

**Figure 1:**
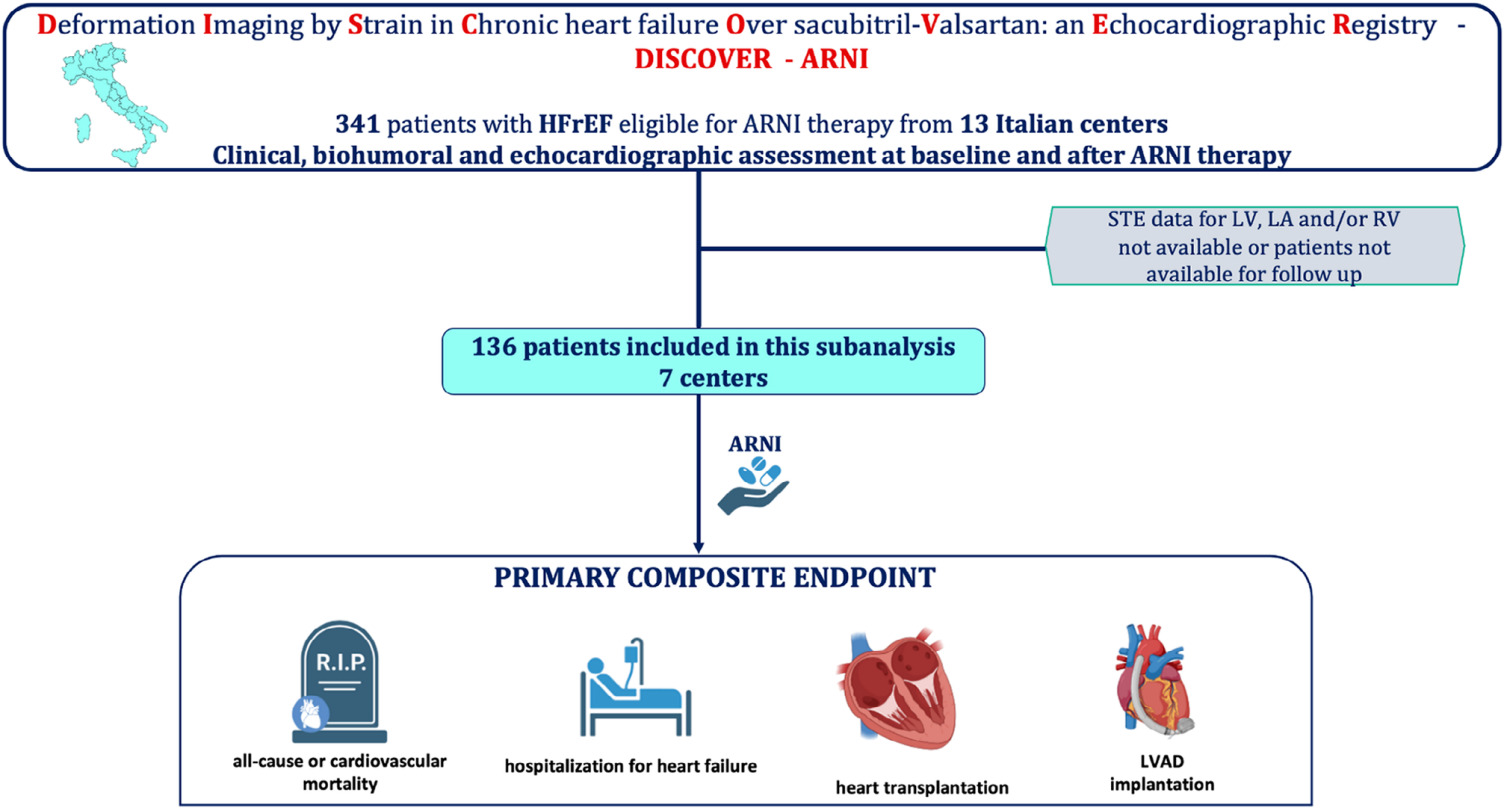
Study design and registered events. ARNI, angiotensin receptor/neprilysin inhibitor; HFrEF, heart failure with reduced ejection fraction; LA, left atrium; LV, left ventricle; LVAD, left ventricular assist devices; RV, right ventricle; STE, speckle tracking echocardiography.

The primary endpoint encompassed a composite of outcomes, including all‐cause or cardiovascular mortality, hospitalization due to HF, heart transplantation, and left ventricular assist device (LVAD) implantation. After a mean follow‐up period of 40 ± 18 months from ARNI initiation, the population was stratified into two groups, based on whether they achieved the primary endpoint or not. Subsequently, we delineated the clinical and echocardiographic data of the sub‐group with available long‐term follow‐up data (*Figure* *1*) and investigated the potential prognostic value of RV strain, acquired before sacubitril/valsartan initiation, as a predictor of the primary endpoint.

## Data collection

The methodology for collecting baseline clinical and echocardiographic data has been extensively described in the first study DISCOVER‐ARNI.^1^ All the study procedures complied with the Declaration of Helsinki. Informed consent was obtained by each patient enrolled.

RV strain was acquired by manually tracing the endocardial border in the four‐chamber apical view, delineating a region of interest (ROI) consisting of six segments for each view. Global RV longitudinal strain (RVLS) was calculated as the mean strain of all RV and interventricular septum segments. Free wall RVLS (fwRVLS) was determined by a ROI of three segments (basal, medial, and apical) encompassing only RV free wall.

## Statistical analysis

Continuous variables were expressed as mean ± standard deviation, if normally distributed, or median [interquartile range] if not normally distributed and were compared using Student's *t*‐test. Kolmogorov–Smirnov test was used to assess variables' normality. Categorical data were expressed as counts and percentages and were compared using the χ^2^‐test. Difference between variables was evaluated by dividing the study cohort into two groups based on the development of composite outcome or not. Multiple events were considered as a single event. Univariate and multivariate analysis by Cox proportional hazard model was employed to investigate the predictive value of strain parameters and other baseline echocardiographic variables that exhibited significant differences among patients who had events or not (excluding variables with missing values >10 and with well‐known collinearity). The optimal cut‐off value of the emerged parameter for the prediction of the primary endpoint was determined using ROC curves, utilizing Youden's index. Subsequently, Kaplan–Meier curves were generated to test the ability of this parameter to stratify the population based on event free survival rates. All analyses were conducted using the Statistical Package for Social Sciences software, version 26.0 (SPSS, Chicago, Illinois), with a *P* value <0.05 considered statistically significant.

## Results

In this sub‐analysis of DISCOVER‐ARNI, we enrolled 136 patients with HFrEF initiating treatment with ARNI. The median age was 65 ± 10 years, with the majority being male (82%). Among them, 76 patients (56%) were classified as New York Heart Association (NYHA) class II, while 54 were NYHA class III, and only one patient was categorized as NYHA class IV (*Table* *1*). Patients presented severe LV dysfunction with a mean left ventricular ejection fraction (LVEF) = 29 ± 6%. At baseline, all STE parameters were reduced in our study cohort, with RV strain showing a slight reduction (LV GLS −8 ± 3%, global PALS 15 ± 6%, fwRVLS −18 ± 5%; *Table* *2*). Ischaemic heart disease was the underlying aetiology of LV disease in 57 patients (42%).

**Table 1:** General characteristics of the study cohort divided into groups based on the development of the primary composite outcome or not

Variables	Overall (*n* = 136)	Events (*n* = 32)	No events (*n* = 104)	*P* value
Age (years)	65 ± 9	66 ± 9	64 ± 10	0.39
Male (%, *n*)	82.4% (112)	93.8% (30)	78.8% (82)	0.05
Height (cm)	168 ± 7	170 ± 5	168 ± 7	0.11
Weight (kg)	79 ± 14	80 ± 11	79 ± 15	0.83
HR (bpm)	68 ± 11	68 ± 10	68 ± 11	0.99
NTproBNP (pg/mL)	1399 ± 2607	1904 ± 3985	1211 ± 1850	0.25
Hypertension (%, *n*)	68.4% (93)	71.9% (23)	67.3% (70)	0.62
Diabetes mellitus (%, *n*)	30.9% (42)	40.6% (13)	27.9% (29)	0.17
Ischaemic aetiology (%, *n*)	41.9% (57)	53.1% (17)	38.5% (40)	0.14
NYHA class				
II	55.9% (76)	50% (16)	57.7% (60)	
III	39.7% (54)	46.9% (15)	37.5% (39)	0.13
IV	0.7% (1)	3.1% (1)	0% (0)	

HR, heart rate; NTproBNP, N‐terminal pro‐brain natriuretic peptide; NYHA, New York Heart Association.

**Table 2:** Echocardiographic characteristics of the study cohort divided into groups based on the development of the primary composite outcome or not

Variables	Overall (*n* = 136)	Events (*n* = 32)	No events (*n* = 104)	*P* value
LV EDV (mL)	184 ± 62	212 ± 65	174 ± 57	**0.002**
LV ESV (mL)	131 ± 52	156 ± 52	122 ± 49	**0.001**
LV EF (%)	29 ± 6	26 ± 5	29 ± 5	**0.006**
E/E′ ratio	15 ± 7	17 ± 9	14 ± 5	0.12
MR > moderate (*n*, %)	40 (29)	10 (31)	30 (28)	0.17
TAPSE (mm)	18 ± 3	17 ± 3	19 ± 3	**0.008**
Tricuspid s′ (cm/s)	9 ± 4	9 ± 4	10 ± 3	0.46
RVFAC (%)	36 ± 9	33 ± 9	38 ± 8	**0.012**
sPAP (mmHg)	32 ± 10	38 ± 11	31 ± 8	**0.001**
RV EDD (mm)	31 ± 6	33 ± 6	31 ± 5	0.059
GLS (%)	−8 ± 3	−7 ± 2	−8 ± 2	**0.002**
PALS (%)	15 ± 6	11 ± 3	15 ± 7	**0.001**
fwRVLS (%)	−18 ± 5	−15 ± 5	−22 ± 5	**0.007**
RV GLS (%)	−14 ± 5	−12 ± 6	−15 ± 4	0.08
∆LV GLS (%)	−1.8 [−2.8; −0.5]	−1.8 [2.8; 11]	−1.8 [−3.3; −0.9]	0.15
∆ Global PALS (%)	3.3 [1.1; 9.6]	7.9	2.3 [1; 7.8]	0.48
∆ fwRVLS (%)	−1.6 [−4.4; −0.1]	−4.8 [−8.8; −0.5]	−1.1 [−0.3; −2.8]	0.06

E/E′, E wave by pulsed‐wave Doppler and E' wave by tissue Doppler imaging ratio; EDD, end‐diastolic diameter; EDV, end‐diastolic volume; ESV, end‐systolic volume; fwRVLS, free wall right ventricular longitudinal strain; LV GLS, left ventricular global longitudinal strain; LV, left ventricular; LVEF, left ventricular ejection fraction; MR, mitral regurgitation; PALS, peak atrial longitudinal strain; RV, right ventricular; RVFAC, right ventricular fractional area change; s′, s′ wave by tissue Doppler imaging; sPAP, systolic pulmonary artery pressure; TAPSE, tricuspid annular plane systolic excursion. Significant *P* values are highlighted in bold.

During a mean follow‐up period of 40 ± 18 months, the combined endpoint was reached by 32 patients (23.5%). Fourteen patients died, mostly due to cardiovascular causes, while twenty‐two were hospitalized for HF, three underwent heart transplantation, and one underwent LVAD implantation (*Figure* *1*).

Baseline demographic, clinical, biohumoural, and echocardiographic characteristics of the study cohort, divided into the two groups based and the presence or absence of events at follow‐up, are summarized in *Tables* *1* and *2*. There were no significant differences observed in general characteristics between the two groups.

Conversely, significant differences were noted in echocardiographic parameters between the two groups: Patients with events exhibited enlarged LV volume and lower LVEF, decreased RV function with tricuspid annular plane systolic excursion (TAPSE) at lower values of normality, and higher systolic pulmonary artery pressure (sPAP). Furthermore, all STE parameters were reduced in patients who experienced events during follow‐up including LV GLS, PALS, and fwRVLS (*Table* *2*).

In a multivariate Cox proportional hazard model incorporating echocardiographic parameters (LVEF, TAPSE, RVFAC, GLS, PALS, and fwRVLS), fwRVLS emerged as the sole independent predictor of the combined endpoint (hazard ratio [HR] 1.15 [1.05; 1.26]; *P* = 0.002) (*Table* *3*). This was confirmed in another multivariate Cox proportional hazard model including LV end‐systolic volume, TAPSE, RVFAC, GLS, PALS and fwRVLS (Table S1).

**Table 3:** Univariate and multivariate analysis by Cox proportional hazard model including left ventricular ejection fraction (LVEF), tricuspid annular plane systolic excursion (TAPSE), right ventricular fractional area change (RVFAC), left ventricular global longitudinal strain (LV GLS), global peak atrial longitudinal strain (PALS), and free wall right ventricular longitudinal strain (fwRVLS) for the prediction of the primary endpoint

Variables	Univariate model HR	*P* value	Multivariate model HR	*P* value
LVEF (%)	0.93	**0.017**	0.48	0.22
TAPSE (mm)	0.95	0.21		
RVFAC (%)	0.92	**0.016**	0.94	0.16
GLS (%)	1.2	**0.03**	1.27	0.25
PALS (%)	0.89	**0.015**	0.54	0.11
fwRVLS (%)	1.2	**0.014**	1.15	**0.002**

Results are expressed as hazard ratios (HRs) per unit increase. Significant *P* values are highlighted in bold.

**Figure 2:**
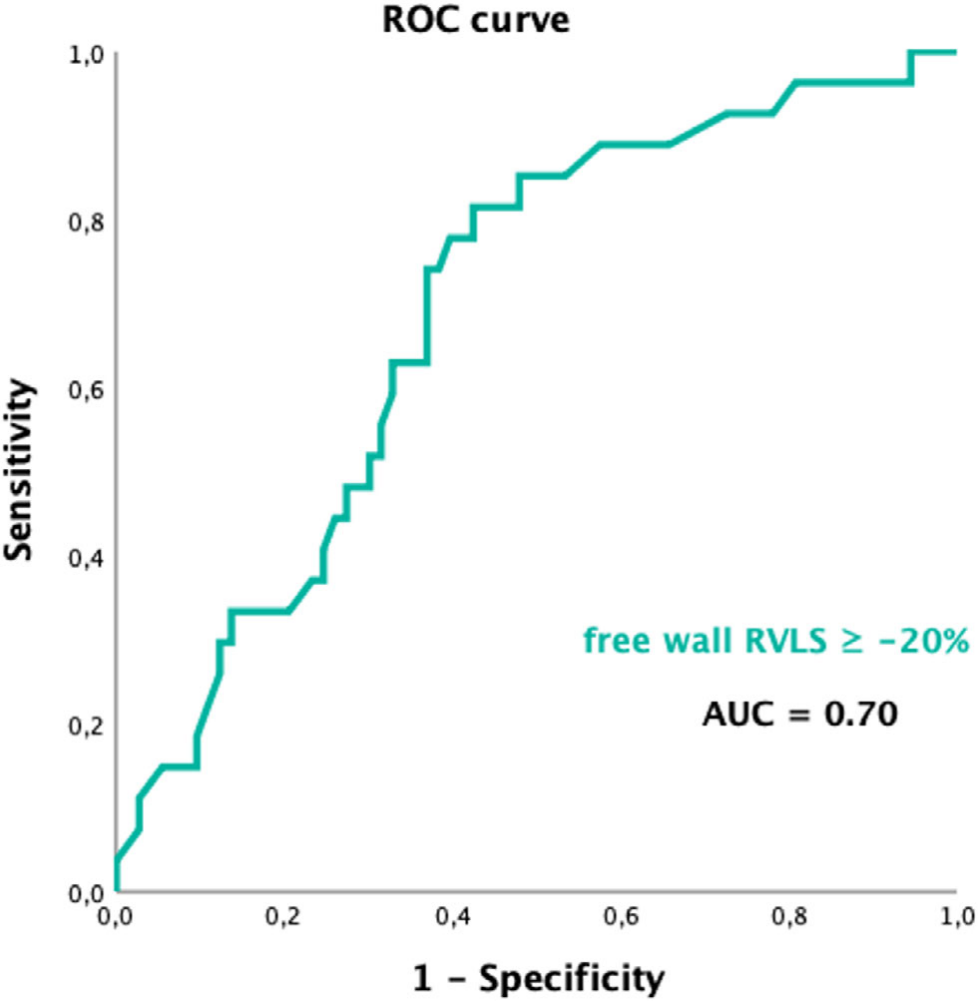
Receiver operating characteristic (ROC) curve of free wall right ventricular longitudinal strain (fwRVLS) for the prediction of the primary endpoint.

**Figure 3:**
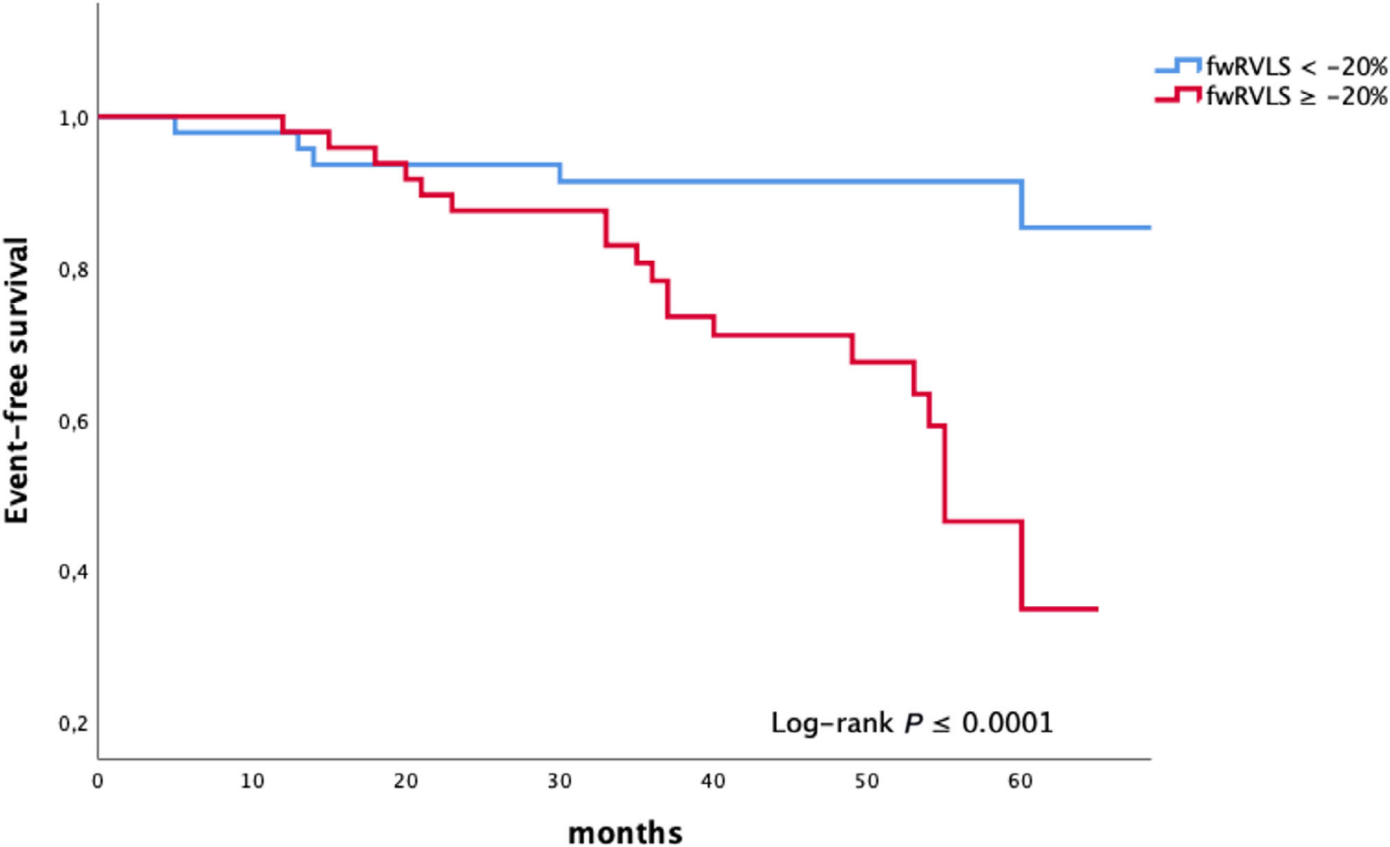
Kaplan–Meier survival curves for composite event‐free survival rate of the study population divided according to free wall right ventricular longitudinal strain (fwRVLS) optimal prognostic cut‐off value.

Using receiver operating characteristic (ROC) curves, the optimal cut‐off value of fwRVLS for predicting the combined endpoint was determined to be −20% with an area under curve (AUC) of 0.70 (*Figure* *2*). A fwRVLS value less negative than −20% was strongly associated with worst prognosis, exhibiting 82% sensitivity and 60% specificity. This cut‐off value was used to construct Kaplan–Meier survival curves, which demonstrated effective risk stratification of fwRVLS ≥ −20% over long‐term follow‐up for overall survival free from primary composite endpoint (*Figure* *3*).

## Discussion

The present study underscores the value of fwRVLS as the only independent predictor of cardiovascular events in patients with HFrEF undergoing sacubitril/valsartan treatment. Particularly, the assessment of fwRVLS prior to ARNI initiation proved to be an independent predictor of the composite endpoint.

To the best of our knowledge, this research represents the first investigation into the prognostic power of fwRVLS in HFrEF patients undergoing ARNI therapy. Several previous studies have highlighted that the feasibility and sensitivity of myocardial contraction measurement by STE are assessing RV contractile function in HFrEF.^8^ Moreover, given its sensitivity in detecting early impairments in systolic function, RV evaluation by STE analysis has shown to surpass conventional echocardiographic parameters (i.e. TAPSE, S′, RVFAC%) as a superior prognostic indicator of clinical outcome in HFrEF patients.^14^, ^16^ In particular, our sub‐analysis revealed that only fwRVLS independently predicted the combined endpoint. This finding is consistent with previous research emphasizing fwRVLS as a more feasible measure of RV function compared to RV GLS.^19^ By focusing on the RV free wall, fwRVLS allows for an assessment of the predominant wall contributing to RV contractile function facilitating an analysis that is feasible and straightforward in clinical practice.^19^, ^20^

Carluccio *et al*.^19^ proposed the clinical significance of incorporating fwRVLS into conventional measurements for patients with HF. In their study, they investigated the prognostic role of fwRVLS in patients with chronic HFrEF and preserved TAPSE for the first time. They found that fwRVLS could aid in reclassifying prognosis and improving risk stratification. Similarly, in our cohort, although the average value of TAPSE at baseline was preserved, albeit at the lower limits of normality in the event groups, baseline fwRVLS was reduced in event group. These findings highlight the importance of utilizing advanced echocardiographic parameters to early assessment of RV dysfunction.

Furthermore, Gavazzoni *et al*.^21^ examined the prognostic value of fwRVLS in outpatients with structural left‐side heart disease without prior hospitalization for HF. Even in this lower‐risk population, fwRVLS remained independently associated with clinical outcomes in the early stages of the disease. These findings suggest that fwRVLS may serve as a valid risk stratification tool applicable across progressive stage of HF.^14^

In this sub‐analysis of DISCOVER‐ARNI, with the advantage of longer and prospective follow‐up, we have delved into the impact of sacubitril/valsartan on clinical outcomes and the potential predictors of treatment success. This adds significant value for clinicians.

Considering the well‐established beneficial effects of sacubitril/valsartan in enhancing survival and clinical outcomes in patients with HFrEF,^4^ it becomes imperative for clinicians to identify baseline indices capable of selecting those patients who are most likely to benefit from ARNI therapy. This allows for an early initiation of treatment, which may be crucial for prognosis. Therefore, our findings suggest that in poorer baseline, echocardiographic parameters, particularly fwRVLS, may obscure RV dysfunction at early stages. As a result, patients may exhibit a lower response to ARNI therapy for long‐term clinical outcome. Early identifying these patients could ensure them to receive advanced HF treatment,^23^ offering potentially greater benefits if administered before over biventricular HF becomes established.

Conversely, patients with less impaired fwRVLS are likely to have a higher probability to recovery with optimized medical treatment and are more likely to avoid unfavourable outcomes. Therefore, we propose the inclusion of this parameter in clinical practice during the echocardiographic examination of these patients. In uncertain scenarios, fwRVLS could serve as an adjunctive tool to guide therapeutic management in patients with HFrEF, facilitating the adjustment of HF medication or the application of advanced therapy.

It was worth noting that RV assessment by STE analysis has demonstrated its utility in selecting patients who would benefit from heart transplantation or LVAD implantation. An efficient prognostic assessment, based on clinical and first and second‐level echocardiographic tools, is essential to optimize these therapeutic resources.^9^ Thus, before LVAD implantation, careful estimation of RV function is required to identify those subjects more likely to experience acute post‐implant RV failure.^12^, ^22^, ^23^ ^,24,25^ Furthermore, among the predictors of prognosis in patients referred for heart transplantation, fwRVLS exhibited the highest predictive values for clinical outcome, even surpassing that of the LV GLS.^24^, ^25^

In the main study DISCOVER‐ARNI,^1^ it was discovered that LV GLS predicted LV reverse remodelling following 6 months of ARNI therapy. Moreover, in the initial sub‐analysis, this parameter served as a predictor of ICD indication within the same cohort and follow‐up period.^18^ These two analyses, conducted retrospectively over a shorter follow‐up duration, focused primarily on LV remodelling data. Conversely, our present study delved into the clinical outcome of the same multicentre cohort over a significant follow‐up period. This extended timeframe allowed us to focus on a more precise definition of the long‐term prognosis. We highlighted the clinical and prognostic significance of incorporating STE parameters, particularly fwRVLS, alongside conventional echocardiographic measurements in assessing patients with HFrEF eligible for ARNI therapy. This comprehensive approach to patient classification may aid in delivering appropriate therapy to enhance clinical outcomes and quality of life, while reducing unnecessary costs deriving from improper treatments, including advanced therapeutic strategies and rehospitalizations.

## Limitations

Despite the promising clinical implications of our study, it is important to acknowledge several limitations. Firstly, the retrospective value led to some missing data, so the study cohort was reduced to 136 compared to the population of DISCOVER‐ARNI, due to challenges upon the quality and non‐focused acoustic window for RV analysis. However, with adequate training, this limitation can be mitigated, and the feasibility of fwRVLS assessment can be enhanced with the introduction of a dedicated software for RV analysis. Moreover, most patients were male, although it is coherent with HF epidemiology, so this may be read as the results of our study were statistically more powerful in male population.

## Conclusions

In the wide panorama of HFrEF treatment, clinical decision‐making and early intervention can present challenges. Advanced echocardiography offers more sensitive parameters for the early identification of biventricular involvement in the disease, which entails poorer prognosis and response to medical treatment. FwRVLS measured by STE emerges as a valuable parameter for evaluating the response to ARNI therapy in terms of overall survival, HF hospitalizations, heart transplantation, or LVAD implantation. This can serve as an adjunctive parameter to guide therapeutic management in patients with HFrEF.

## Conflict of interest

The authors have no conflict of interest to declare.

## Fundings

None.

## Supporting information


**Table S1** Univariate and multivariate analysis by Cox Proportional Hazard Model including left ventricular end‐systolic volume (LVESV), tricuspid annular plane systolic excursion (TAPSE), right ventricular fractional area change (RVFAC), left ventricular global longitudinal strain (LV GLS), global peak atrial longitudinal strain (PALS), free wall right ventricular longitudinal strain (fwRVLS) for the prediction of the primary endpoint. *Results are expressed as hazard ratios (HRs) per unit increase*.

## Data Availability

The data underlying this article will be shared on reasonable request to the corresponding author.
