# Supplementary material for: Right ventricular strain predicts outcome in patients receiving sacubitril/valsartan: A sub‐analysis of DISCOVER‐ARNI
Source: ESC Heart Fail. 2025 Apr 16;12(4):2878–86. doi: 10.1002/ehf2.15297 (PMC12287863; doi:10.1002/ehf2.15297)
Supplement: Supplementary file 1 — Table S1 Univariate and multivariate analysis by Cox Proportional Hazard Model including left ventricular end‐systolic volume (LVESV), tricuspid annular plane systolic excursion (TAPSE), right ventricular fractional area change (RVFAC), left ventricular global longitudinal strain (LV GLS), global peak atrial longitudinal strain (PALS), free wall right ventricular longitudinal strain (fwRVLS) for the prediction of the primary endpoint. Results are expressed as hazard ratios (HRs) per unit increase. [file EHF2-12-2878-s001.docx]

**Supplementary Table 1 .** Univariate and multivariate analysis by Cox Proportional Hazard Model including left ventricular end-systolic volume (LVESV), tricuspid annular plane systolic excursion (TAPSE), right ventricular fractional area change (RVFAC), left ventricular global longitudinal strain (LV GLS), global peak atrial longitudinal strain (PALS), free wall right ventricular longitudinal strain (fwRVLS) for the prediction of the primary endpoint. *Results are expressed as hazard ratios (HRs) per unit increase.*

| **Variables** | **Univariate model HR** | **p-value** | **Multivariate model HR** | **p-value** |
| --- | --- | --- | --- | --- |
| **LV ESV (%)** | 1.01 | **0.002** | 6.18 | 0.13 |
| **TAPSE (mm)** | 0.95 | 0.21 |  |  |
| **GLS (%)** | 1.2 | **0.03** | 1.27 | 0.25 |
| **PALS (%)** | 0.89 | **0.015** | 0.54 | 0.11 |
| **fwRVLS (%)** | 1.2 | **0.014** | 1.15 | **0.002** |
